# Effect of scalp nerve block with ropivacaine on postoperative pain in pediatric patients undergoing craniotomy: A randomized controlled trial

**DOI:** 10.3389/fmed.2022.952064

**Published:** 2022-09-07

**Authors:** Li Ning, Lai Jiang, Qingqing Zhang, Mengqiang Luo, Daojie Xu, Yuanzhi Peng

**Affiliations:** ^1^Department of Anesthesiology and Surgical Intensive Care Unit, Xinhua Hospital, Shanghai Jiaotong University School of Medicine, Shanghai, China; ^2^Department of Anesthesiology, Huashan Hospital, Fudan University, Shanghai, China

**Keywords:** scalp nerve block, postoperative pain, pediatric craniotomy, hemodynamic stability, ropivacaine

## Abstract

**Background:**

Scalp nerve block (SNB) is widely used for postoperative pain control, intraoperative hemodynamic control, and opioid-sparing in adult craniotomies. However, there are few studies of SNB in pediatric patients undergoing craniotomy. In the present study, we aimed to investigate the effect of SNB on postoperative pain, intraoperative hemodynamic stability, and narcotic consumption in pediatric craniotomy under general anesthesia.

**Methods:**

This trial is a single-center, prospective, randomized, and double-blind study. A total of 50 children aged between 2 and 12 years who are undergoing elective brain tumor surgery will be randomly allocated in a 1:1 ratio to receive either 0.2% ropivacaine for SNB (group SNB, intervention group, *n* = 25) or the same volume of saline (group Ctrl, control group, *n* = 25). The primary outcome was to assess the score of postoperative pain intensity at time 1, 4, 8, 12, 24, and 48 h postoperatively using the FLACC score method. Secondary outcomes were to record intraoperative hemodynamic variables (MAP and HR) during skull-pin fixation, skin incision and end of skin closure, intraoperative total consumption of remifentanil and propofol, postoperative opioid consumption, and the incidence of postoperative nausea and vomiting.

**Results:**

Fifty patients were analyzed (*n* = 25 in SNB group; *n* = 25 in control group). Compared to the control group, postoperative pain intensity was significantly relieved in the SNB group up to 8 h post-operatively. In addition, SNB provided good intraoperative hemodynamic stability, reduced intraoperative overall propofol and remifentanil consumption rate, and postoperative fentanyl consumption compared to the control group. However, the incidence of postoperative nausea and vomiting was not different between SNB and the control group.

**Conclusions:**

In pediatric craniotomies, SNB with 0.2% ropivacaine provides adequate postoperative pain control and good intraoperative hemodynamic stability during noxious events compared to the control group.

**Clinical trial registration:**

Chinese Clinical Trial Registry [No: ChiCTR2100050594], Prospective registration.

## Introduction

For a long time, postoperative pain has not received sufficient attention in pediatric craniotomy patients (1, 2). On the one hand, young children, particularly infants, cannot properly describe their pain and it is sometimes difficult to distinguish painful or emotional responses in young children, so the pain after surgery is often inappropriately considered associated with emotional responses (3–5). On the other hand, many neurosurgeons fear that the use of opioids may interfere with neurologic examination (6). Moreover, opioid-induced side effects, such as nausea, vomiting, and especially respiratory depression may lead to disastrous results (7, 8). Therefore, prevention and treatment of postoperative pain in pediatric craniotomy patients is still a challenging clinical problem.

Inadequate postoperative pain control in children following craniotomy may cause severe consequences such as agitation, intracranial hypertension, and postoperative hemorrhage, which may increase morbidity and mortality (9, 10). Given the side effects of opioids, it is necessary to minimize reliance on opioid analgesia in craniotomy patients. Multimodal analgesia which combines low doses of systemic analgesics with local anesthetics for scalp infiltration or regional scalp nerve block has been proposed to prevent postoperative pain in adult craniotomy patients (11–13). However, the optimal postoperative analgesic management for pediatric craniotomy patients remains elusive.

SNB has been widely used as the principal anesthetic in awake craniotomies or served as an adjuvant method to general anesthesia in adult supratentorial craniotomies (13, 14). SNB can attenuate postoperative pain, decrease opioid and narcotic agent consumption and prevent intraoperative hemodynamic responses to noxious stimulation (12, 15). However, over the past decade, the studies of SNB mostly focused on adult craniotomy patients, and there are few studies of SNB in pediatric patients undergoing craniotomy.

The purpose of our study was to determine if SNB with ropivacaine reduce postoperative pain score in pediatric patients undergoing craniotomy. Our primary hypothesis was that SNB before surgery would improve postoperative pain control, and the secondary hypothesis was that SNB would provide intraoperative hemodynamic stability, and reduce perioperative opioid and narcotic agent consumption.

## Materials and methods

### Participants

This prospective, randomized, placebo-controlled, double-blind study was approved by the Institutional Ethical Committee of Xinhua Hospital, Medical School, Shanghai Jiaotong University, and was registered in the Chinese Clinical Trial Registry (registration number: ChiCTR2100050594). Written informed consent was obtained prior to study enrollment. Pediatric patients aged 2–12 years presenting for elective supratentorial craniotomies will be recruited from Xinhua Hospital, Medical School, Shanghai Jiaotong University from July 2020 to October 2021.

### Patient recruitment and assignment to groups

Sixty pediatric patients aged 2–12 years with American Society of Anesthesiologists (ASA) physical status I or II scheduled to undergo elective supratentorial brain tumor surgery and to receive general anesthesia were prospectively screened for possible inclusion. The exclusion criteria included: (1) Pediatric patients aged>12 or <2 years; (2) Children with mental disorders; (3) Children whose authorized surrogates are unwilling to participate in the study; (4) Children with severe diseases or cardiac insufficiency; (5) Emergency craniotomies; (6) Children with severe kidney or liver diseases; (7) Children with severe coagulation disorders; (8) Children who cannot be weaned from endotracheal intubation following surgery; (9) Children with a history of allergy to opioids or other anesthetics; (10) Children with a history of analgesic substance abuse.

### Randomization

After meeting the eligibility criteria and signing the informed consent to participate in the study, Patients were randomized in a 1:1 ratio into two groups using computer-generated randomized numbers. Groups differed according to the performance of a SNB with either 0.2% ropivacaine (Group SNB, intervention group) or the same volume of saline (Group Ctrl, control group). The SNB was performed by the attending anesthesiologists who were blinded to the agents which has been prepared by a nurse non-involved in the study in identical syringes. Patients, children's guardians, anesthesiologists, and neurosurgeons were blind to group assignment. The outcome assessors who were blinded to randomization and did not participate in anesthetic management and data recording or analysis. All of them received the use of evaluation scale training and recorded pain scores and complications postoperatively.

### Anesthesia and analgesia

All patients received standardized anesthetic monitoring including non-invasive blood pressure (BP), heart rate (HR), pulse oximetry saturation (SpO_2_), invasive arterial pressure, end-tidal carbon dioxide partial pressure (P_ET_CO_2_), and anesthesia gas monitoring. General anesthesia was induced with intravenous atropine (0.01 mg/kg), midazolam (0.1 mg/kg), propofol (2–3 mg/kg), fentanyl (1–2 μg/kg), and cisatracurium (0.2 mg/kg). In addition, dexamethasone (0.2 mg/kg to a maximum dose of 5 mg) was given after anesthesia induction. Following anesthesia induction, continuous invasive blood pressure monitoring was established through a radial arterial catheterization, and a jugular vein catheter was inserted. Mechanical ventilation was performed in all patients (50% air in oxygen) to maintain an O_2_ saturation of >98% and an end-expiratory CO_2_ of 30–35 mmHg.

Anesthesia was maintained with 0.5 MAC (minimum alveolar concentration) sevoflurane at an inhalational concentration of 1–1.5% and an intravenous infusion with propofol 3–6 mg/kg/h and remifentanil 0.05–0.25 μg/kg/min. Cisatracurium was administered intraoperatively as needed and was reversed at the end of the surgery with neostigmine (0.04 mg/kg) and atropine (0.015 mg/kg). Mean arterial blood pressure and heart rate were maintained within 20% of baseline measures. Remifentanil was adjusted by steps of 0.05–0.1 μg/kg/min if intraoperative MAP or HR over or below 20% of baseline values. If the adjustment failed, propofol was adjusted by steps of 0.5–1 mg/kg/h until stabilization within the 20% range. Baseline values were defined as 3-min averaged values immediately before the performance of SNB. Fentanyl (1 μg/kg) was given 20 min before the end of surgery. Additionally, all patients received tropisetron hydrochloride (0.2 mg/kg to a maximum dose of 5 mg) as an emesis prophylaxis 30 min before the end of surgery. No other intraoperative adjuvant analgesics were given.

After surgery, mechanical ventilation was discontinued, and the patient was ready for tracheal extubation when consciousness and sufficient spontaneous breathing recovered and hemodynamics was stable. All the patients were equipped with nurse-controlled intravenous analgesia (NCIA) device containing a fentanyl solution (15 μg/kg fentanyl, 0.2 mg/kg dexamethasone to a maximum dose of 5 mg and 0.2 mg/kg tropisetron hydrochloride to a maximum dose of 5 mg, the total volume was diluted to 100 ml with 0.9% normal saline). The NCIA device was connected to the IV line before the end of surgery (parameters: background dose-rate: 2 ml/h; boluses: 2 ml, 15 min refractory time). If the score of postoperative pain <3, the Acute Pain Services who were blinded to study treatment allocation will consider discontinuing the NCIA. The care physicians or nurses had been informed of the adequate NCIA use the day before surgery and blinded to randomization.

### Regional scalp block

Bilateral scalp nerve blocks were performed by the anesthesiologist before skull-pin fixation and after induction of general anesthesia. The block was performed by the attending anesthesiologists who were blinded to the agents. The anesthetic solution was prepared by a nurse, who was not participating in patient anesthetic management and data recording or analysis, according to the computer-generated randomization list. For SNB group patients, the syringe was containing 20 mL of 0.2% ropivacaine. For control group patients, the syringe was containing 20 mL of 0.9% normal saline.

Bilateral scalp nerve blocks were done at several points over the scalp. (1) Bilateral supraorbital nerve (1–2 ml); (2) Bilateral supratrochlear nerves (1–2 ml); (3) Bilateral zygomatic temporal nerve (1–2 ml); (4) Bilateral auriculotemporal nerve (1–2 ml); (5) Bilateral greater occipital nerve (2–3 ml); (6) Bilateral lesser occipital nerve (2–3 ml).

### Outcomes

The primary measured outcome was the score of postoperative pain which was assessed at time 1, 4, 8, 12, 24, and 48 h postoperatively. In our study, postoperative pain was evaluated by the Faces, Legs, Activity, Cry, and Consolability Scale (FLACC, 0–10 scores) following pediatric surgery (16). Secondary outcomes included: the intraoperative hemodynamic variables (MAP and HR) during baseline, skull-pin fixation, skin incision, and end of skin closure; The overall consumption rate of propofol (mg/kg/h) and remifentanil (μg/kg/min); The total amount of fentanyl consumption and the total number of compressions of NCIA device within 48 h postoperatively; The incidence of postoperative nausea and vomiting (PONV) and complications both from local anesthetic and the nerve block were also assessed.

### Sample size calculation

The sample size was calculated based on data available in published studies and our clinical experience at the study centers using the G^*^ Power software (version 3.1.9.2, Franz Faul, Kiel University, Germany) (9, 10). According to previous studies, the incidence of moderate postoperative pain in children is ~50% in pediatric craniotomy patients (10). Assuming a two-sided α value of 0.05 and a β value of 0.2, we estimated that 21 patients in each group would be required to detect a 1.8-point difference in the FLACC score. Considering a 20% dropout rate, 27 patients were recruited in each group. Therefore, the total sample size was 54 patients in this study.

### Statistical analyses

SPSS version 22.0 (International Business Machines Inc.) was used for data analysis. All analyses were conducted using the modified intention-to-treat principle. All data are either presented as median (IQR) or mean (SD), or as frequency and percentage (%), respectively. Categorical variables (sex, ASA physical status) were presented as frequencies and percentages. The chi-square test was used for comparing proportions, the Shapiro–Wilk test was used to test the normal distribution of continuous variables. Student's *t*-test was used to compare normally distributed outcome variables between the two randomized groups. MAP and HR were compared using 2-way mixed-design analysis of variance (ANOVA) and Tukey's multiple comparisons test for *post-hoc* comparisons. FLACC score was compared using the Mann-Whitney U test for non-parametric variables (17, 18). Two-tailed analyses were conducted, and a *p* ≤ 0.05 was considered statistically significant.

## Results

### Demographic characteristics

**Figure 1:**
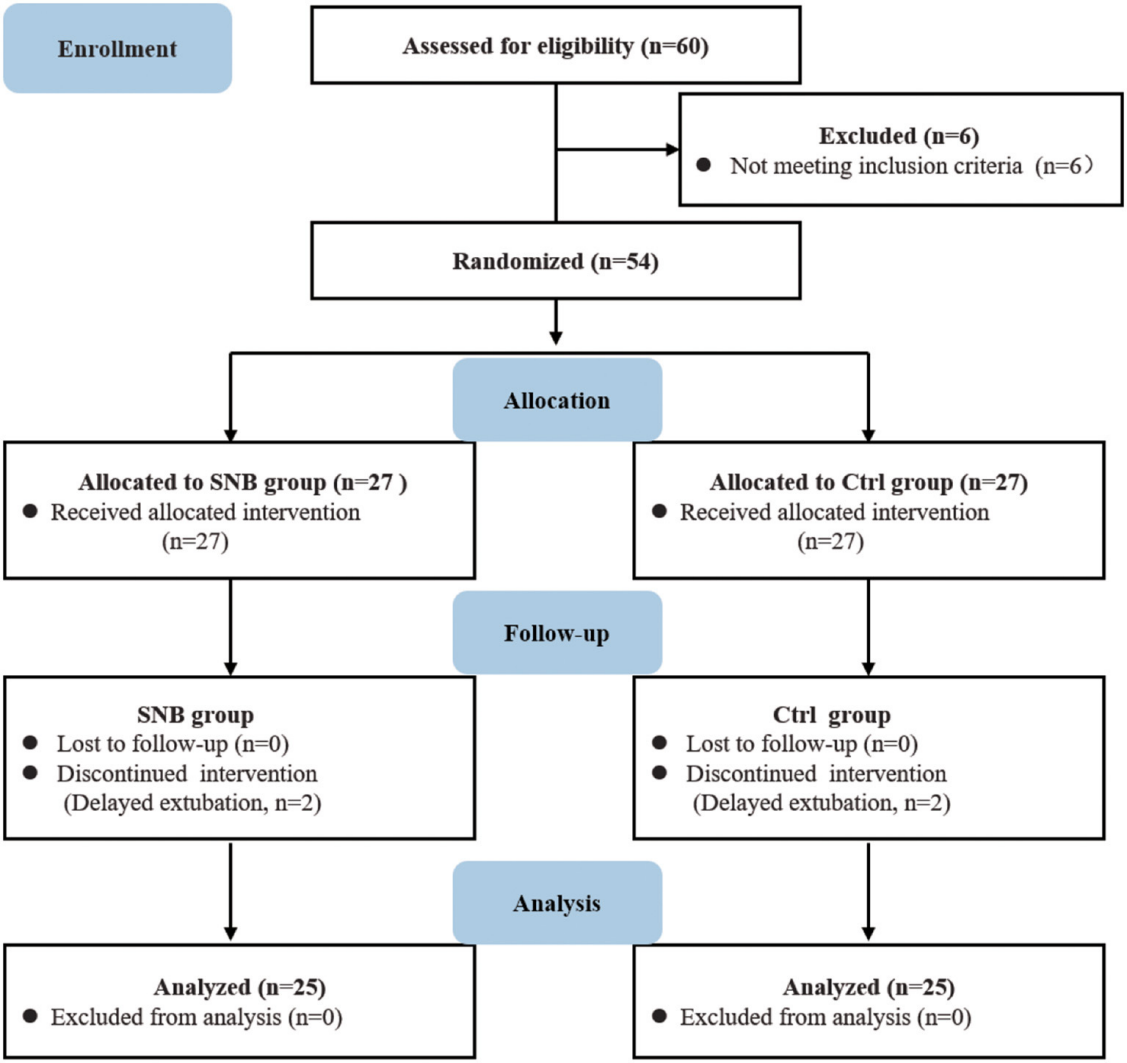
CONSORT diagram showing patient recruitment and follow-up. group Ctrl, control group; group SNB, scalp nerve block group.

As in Figure 1, a total of 60 patients who underwent major craniotomy were enrolled in the study, of whom 54 patients were randomized into group control and group SNB, with 27 patients in the group control and 27 patients in the group SNB (Figure 1). Among them, two patients in the group control and two patients in the group SNB were excluded from analysis for delaying extubation postoperatively, the outcomes in those patients could not be assessed. Ultimately, 50 patients were completed in this study. There were no significant differences between group control and group SNB in demographic characteristics of patients and operative variables, including sex, age, weight, ASA status, the duration of operation and anesthesia, recovery time from anesthesia (Table 1, *P* > 0.05).

**Table 1:** Demographic characteristics of group Ctrl and group SNB.

**Characteristics**	**Ctrl** **(*n* = 25)**	**SNB** **(*n* = 25)**	***P*-value**
Sex (female/male)	9/16	9/16	1.00
Age (year)	6.36 ± 2.99	6.72 ± 3.42	0.69
Weight (kg)	26.39 ± 11.42	26.68 ± 12.84	0.93
ASA physical status [1/2, *n* (% 1)]	10/15 (40.0)	11/14 (44.0)	1.00
Duration of operation (h)	4.83 ± 1.22	4.43 ± 1.37	0.28
Duration of anesthesia (h)	5.43 ± 0.79	5.12 ± 1.27	0.35
Time for recovery from anesthesia (min)	12.20 ± 3.51	11.92 ± 4.09	0.79

Data are presented as mean ± SD or percentage (%) as indicated. ASA, American Society of Anesthesiologists; group Ctrl, control group; group SNB, scalp nerve block group.

### Primary outcome

Pain intensity was evaluated at 1, 4, 8, 12, 24, and 48 h after surgery. FLACC pain scores were significantly decreased in the SNB group compared to the control group at postoperative 1, 4, and 8 h (Table 2, *P* < 0.05). The pain intensity gradually decreased after postoperative 8 h. However, there was no significant difference at postoperative 12, 24, and 48 h between the two groups (Table 2, *P* > 0.05).

**Table 2:** Pain scores across postoperative time points.

**FLACC scores (h)**	**Ctrl** **(*n* = 25)**	**SNB** **(*n* = 25)**	***P*-value**
1	2 (2–3)	1 (0–1.5)	<0.001
4	4 (4–5)	2 (2–3)	<0.001
8	5 (4–6)	3 (2.5–4.5)	<0.01
12	4 (3–5)	3 (2–4.5)	>0.05
24	3 (2–5)	3 (1–4)	>0.05
48	2 (0.5–3)	2 (0–3)	>0.05

Data are presented as median (IQR). group Ctrl, control group; group SNB, scalp nerve block group; FLACC, face, legs, activity, cry, and consolability scale.

### Secondary outcomes

**Figure 2:**
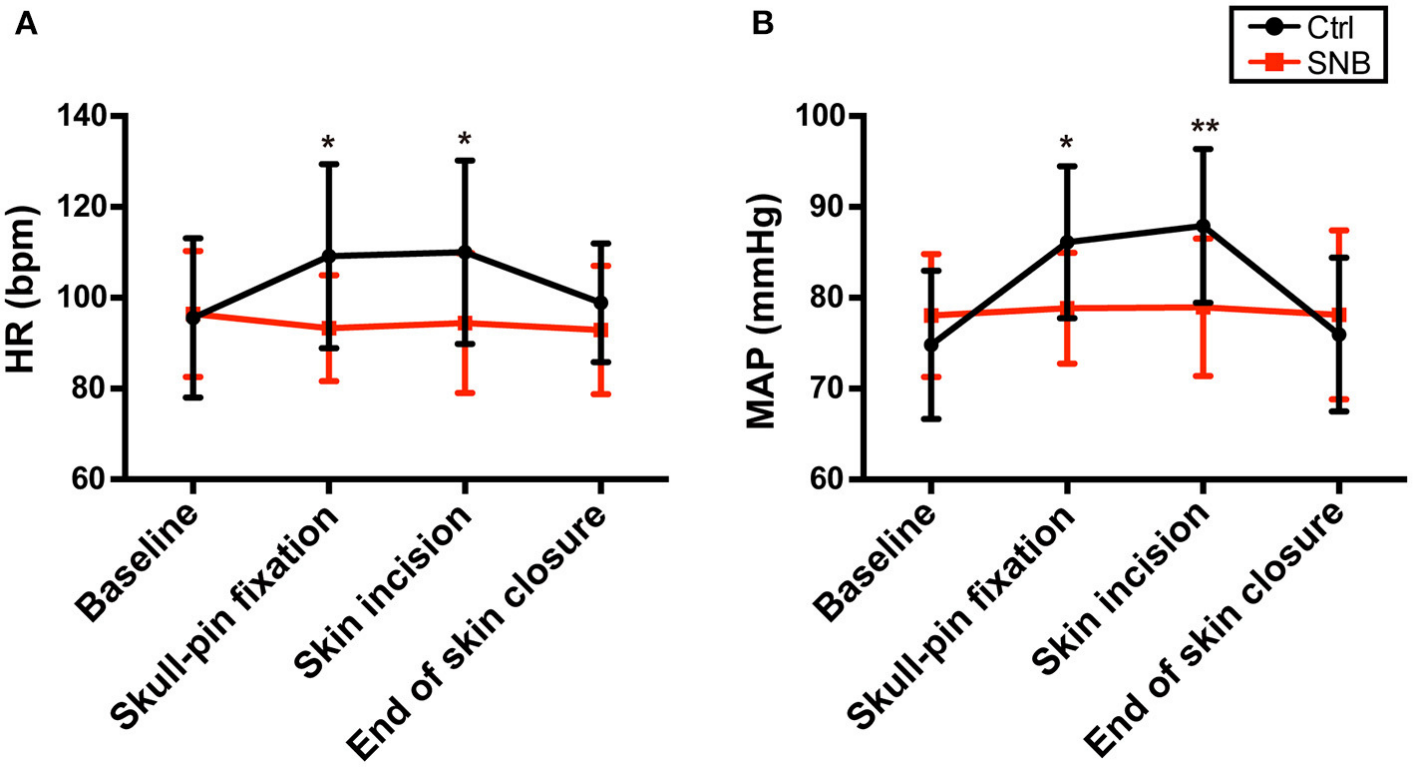
The intraoperative hemodynamic variables (HR and MAP) in group Ctrl and group SNB. Changes in HR **(A)** and MAP **(B)** during baseline, skull-pin fixation, skin incision, and end of skin closure in group Ctrl and group SNB. MAP, mean arterial blood pressure; HR, heart rate. Data are presented as mean ± SD. *P < 0.05; **P < 0.01. group Ctrl, control group; group SNB, scalp nerve block group.

The MAP and HR were significantly higher in group control than in group SNB at the time of skull-pin fixation and skin incision (Figure 2, *P* < 0.05). However, there was no significant difference at the time of end of skin closure between two groups (Figure 2, *P* > 0.05).

**Figure 3:**
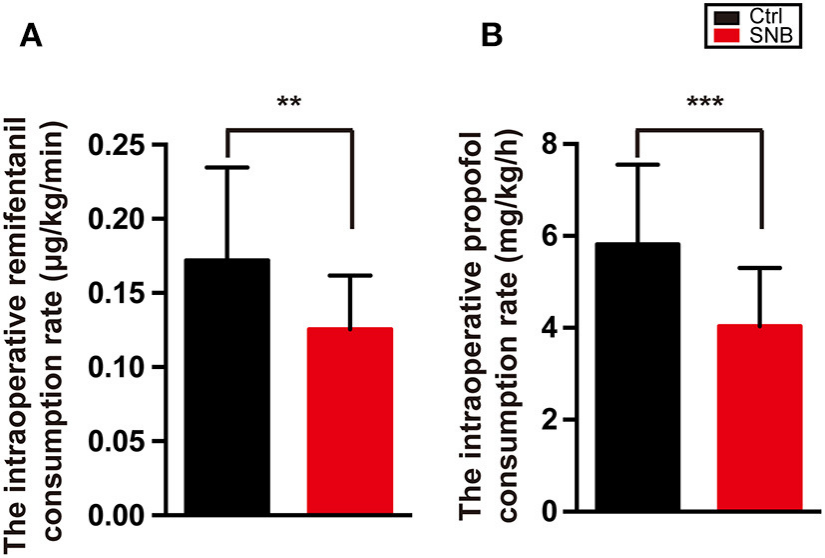
The intraoperative total remifentanil **(A)** and propofol **(B)** consumption rate in group Ctrl and group SNB. Data are presented as mean ± SD. **P < 0.01; ***P < 0.001. group Ctrl, control group; group SNB, scalp nerve block group.

**Figure 4:**
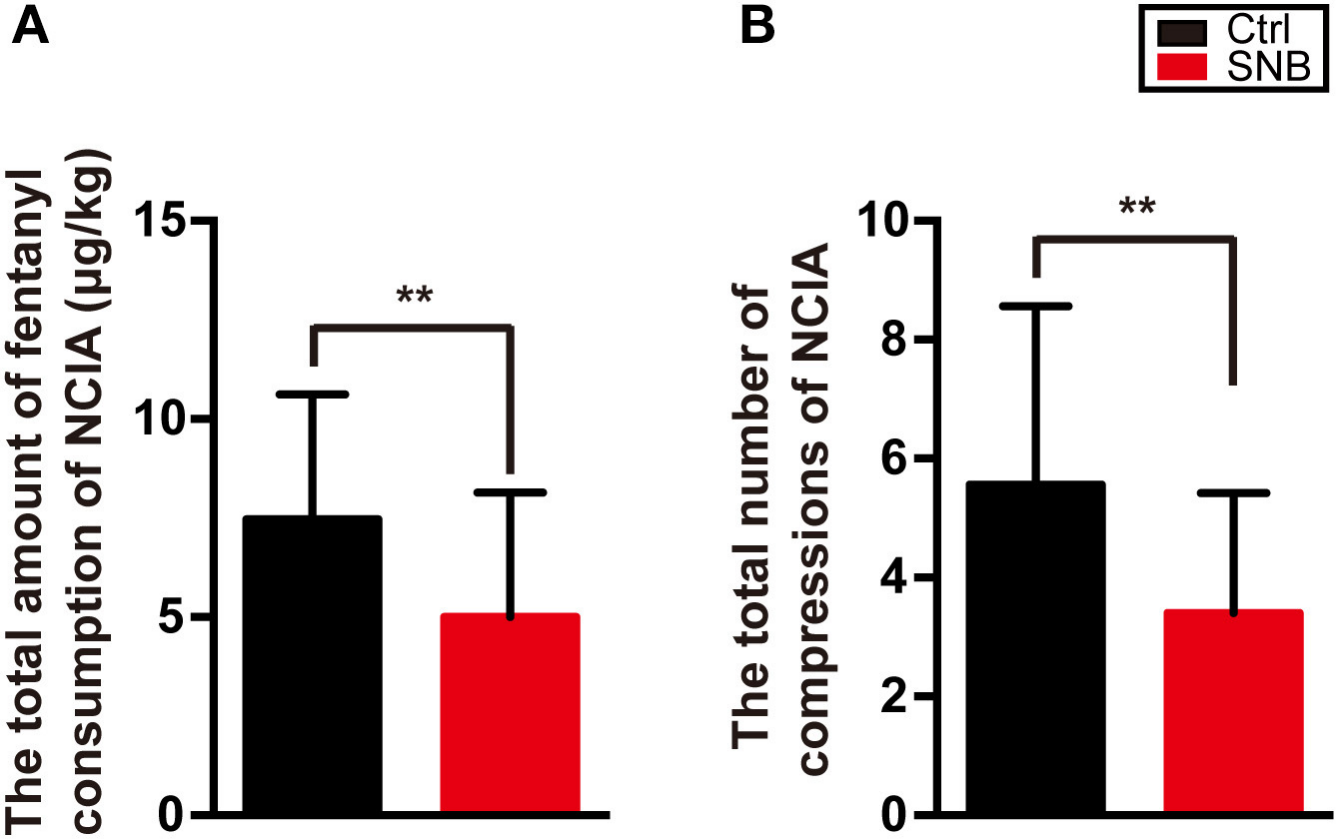
The total amount of fentanyl consumption **(A)** and the total number of compressions **(B)** of NCIA in group Ctrl and in group SNB within postoperative 48 h. Data are presented as mean ± SD. **P < 0.01. NCIA, nurse-controlled intravenous analgesia; group Ctrl, control group; group SNB, scalp nerve block group.

The overall intraoperative consumption rate of propofol and remifentanil was significantly higher in group control than in group SNB (Figure 3, *P* < 0.001). The total amount of medicine used in the postoperative analgesia pump was calculated. The total amount of fentanyl consumption and the total number of compressions of NCIA were significantly higher in group control than in group SNB during postoperative 48 h (Figure 4, *P* < 0.001).

There was no statistical difference in the incidence of PONV between the two groups (Table 3, *P* > 0.05). In our study, no adverse effects such as post-operative scalp infection, hematoma, or local anesthetic toxicity were observed in patients during the study period (Table 3, *P* > 0.05).

**Table 3:** The incidence of PONV, local anesthetic toxicity, and SNB complications.

**Complications**	**Ctrl** **(*n* = 25)**	**SNB** **(*n* = 25)**	***P*-value**
Postoperative nausea and vomiting within 48 h	10/25	11/25	1.00
Local anesthetic toxicity	0/25	0/25	1.00
Post-operative scalp infection or hematoma	0/25	0/25	1.00

Data are presented as the total number of patients (n). PONV, postoperative nausea and vomiting; group Ctrl, control group; group SNB, scalp nerve block group.

## Discussion

In the present study, we found that bilateral SNB with ropivacaine improved postoperative pain control for up to 8 h compared to the control group in pediatric craniotomy patients. Furthermore, bilateral SNB provided good intraoperative hemodynamic stability and reduced propofol and opioid consumption compared to the control group.

Regional anesthesia is mainly used to provide postoperative analgesia. Preemptive analgesia with local anesthetics is an effective method of postoperative pain control (13). The theory was that preemptive analgesia prior to surgery can prevent central sensitization caused by noxious stimuli and inflammation (19, 20). Previous studies demonstrated that preoperative SNB has beneficial to postoperative pain in adult craniotomy patients (12, 13). In our study, we found that SNB with 0.2% ropivacaine provided preferable analgesia which relieved postoperative pain for up to 8 h postoperatively. The previous studies showed the persistent analgesic time of SNB on postoperative pain was 4–48 h in adult craniotomy patients (12, 15). The reasons for the different duration of SNB on postoperative pain may be related to the type of local anesthetic, the dose of local anesthetic, and the compound application of epinephrine. In the present study, we did not add epinephrine for SNB. In light of the mean duration of operation was over 4 h in scalp nerve block group, the persistent analgesic time of SNB on postoperative analgesia was up to 8 h. Therefore, it is supposed to be at least 12 h to return the sensitivity of the scalp nerve, which was far longer than the duration of action of ropivacaine (about 3 h). This long-lasting effect on postoperative analgesia might be due to preemptive analgesia.

Hemodynamic stabilization is important to neurosurgery patients both in the intraoperative and postoperative periods. The elevation of blood pressure may cause an abrupt increase of intracranial pressure and favor bleeding in injured parenchyma with fragile hemostasis (21, 22). In the absence of regional anesthesia, deep anesthesia is usually used to control hemodynamic variations in response to noxious stimulation, including an increase in opioid and/or hypnotic anesthetic agent concentrations (23–25). However, too deep anesthesia may cause deleterious consequences such as hypotension, bradycardia, and increase postoperative morbidity and mortality (26, 27). In our study, we found that the hemodynamics were stable during dramatic noxious stimulation in the SNB group compared to the control group. Moreover, SNB decreased the intraoperative propofol and remifentanil total consumption, consistent with the previous studies in adult craniotomy patients (12, 15).

Another advantage of combining SNB with general anesthesia in pediatric craniotomy patients has the potential for decreasing intraoperative general anesthetic requirements. Pediatric neurosurgery usually takes a long time, so it requires a lot of general anesthetics in the absence of regional anesthesia (9, 10). However, the neurotoxicity of general anesthetics have been well-documented in some animal models and clinical trials (28–30). The dose of general anesthetics is an important factor in neurotoxicity in the developing brain (31). Therefore, it is necessary to pay attention to the neuron death of general anesthetics exposure in pediatric craniotomy patients. In the present study, combining SNB with general anesthesia significantly decreased intraoperative general anesthetic consumption. An eventual beneficial effect of SNB on neurological prognosis should be the object of a specifically designed study.

Postoperative nausea and vomiting (PONV) are the common and distressing symptoms after craniotomy. PONV may not only generate lower patient satisfaction but cause deleterious consequences such as intracranial hypertension and postoperative intracranial hemorrhage (32). In light of opioids being a risk factor for PONV, the use of regional anesthesia and non-opioid analgesics has been proposed to decrease PONV (33, 34). In our study, opioid consumption was decreased both in the intraoperative and postoperative periods. However, the incidence of PONV had no significant difference between the two groups within 48 h postoperatively. Although we used dexamethasone and tropisetron hydrochloride intraoperative for PONV prophylaxis, the incidence of PONV was up to 42% (10 patients in the control group and 11 patients in the SNB group) within the first 48 h after surgery. The high incidence of PONV in our study might be related to the duration of surgery, use of volatile anesthetic agents, brain tumor size, and postoperative intracranial pressure.

Ropivacaine is widely used in pediatric regional anesthesia for its minimal cardiotoxicity and neurotoxicity (35). Additionally, ropivacaine has a relatively short onset time and long effect duration as compared to bupivacaine and lidocaine, respectively (36). It has become one of the most popular local anesthetics for pediatric regional blocks (36, 37). In our study, we used 0.2% ropivacaine for SNB and the total dosage of ropivacaine did not exceed 2 mg/kg. The concentration was demonstrated safety in pediatrics which the concentration of ropivacaine was 0.1–0.375% and the total dosage was should not exceed 3 mg/kg for caudal blocks (37, 38).

Our study has several limitations. First, we only examined the effect of 0.2% ropivacaine on postoperative pain and did not examine the effect of other concentrations of ropivacaine on postoperative pain. Second, we did not examine the plasma concentration of ropivacaine in our patients to rule out the risk of toxicity with our SNB technique. Because the scalp is rich in blood vessels with rapid local anesthetic uptake, local anesthetic injection may predispose to local anesthetic toxicity (39). However, we did not find any local anesthetics toxicity of 0.2% ropivacaine for SNB in our study. Third, epinephrine was recommended in well-vascularized areas to maximize block duration and minimize acute rises in anesthetic plasma concentration (40). In the present study, we did not supplement with epinephrine during SNB. It is possible to prolong the postoperative analgesia time by adding epinephrine. Fourth, postoperative pain was assessed by the FLACC in our study, which was commonly used for the evaluation of postoperative pain aged 1–18 years for hospitalized children (16). Although children older than 7 years of age can use NRS or VAS for self-assessment, some children were apathetic after neurosurgery, so the reliability may decline through self-assessment methods such as NRS and VAS in children after neurosurgery.

SNB has been proposed as an adjuvant to general anesthesia for postoperative pain control, intraoperative hemodynamic control, and opioid-sparing in adult craniotomy. However, the SNB technique is not commonly used in pediatric craniotomy patients so far. In the present study, we demonstrated that SNB combined with general anesthesia significantly improved postoperative pain control, intraoperative hemodynamic control, and reduced opioid consumption in pediatric craniotomy patients compared to the control group. It was no adverse effects with 0.2% ropivacaine for SNB pediatric craniotomy patients. Therefore, the SNB is a safety technique in pediatric craniotomy patients.

## Data availability statement

The original contributions presented in the study are included in the article/supplementary material, further inquiries can be directed to the corresponding author/s.

## Ethics statement

The studies involving human participants were reviewed and approved by Institutional Ethical Committee of Xinhua Hospital, Medical School, Shanghai Jiao Tong University. Written informed consent to participate in this study was provided by the participants' legal guardian/next of kin.

## Author contributions

LN designed, planned, performed the experiments, analyzed the data, and contributed to writing the manuscript. YP designed, planned, performed the experiments, and analyzed the data. DX designed the experiments and wrote the manuscript. ML designed the experiments. LJ oversaw the research phases. QZ assisted in completing the experiments. All authors contributed to the article and approved the submitted version.

## Funding

Funding was provided by Xinhua Hospital, Shanghai Jiao Tong University School of Medicine (Grant No. 2021XHYYJJ08).

## Conflict of interest

The authors declare that the research was conducted in the absence of any commercial or financial relationships that could be construed as a potential conflict of interest.

## Publisher's note

All claims expressed in this article are solely those of the authors and do not necessarily represent those of their affiliated organizations, or those of the publisher, the editors and the reviewers. Any product that may be evaluated in this article, or claim that may be made by its manufacturer, is not guaranteed or endorsed by the publisher.
